# Draft-genome sequence data and phylogenomic comparison of two marine-sourced bacterial strains *Pseudoalteromonas sp*. MIP2626 and *Psychrobacter sp*. BI730

**DOI:** 10.1016/j.dib.2020.105898

**Published:** 2020-06-25

**Authors:** Caroline Isabel Kothe, Christine Delbarre-Ladrat, Pierre Renault, Delphine Passerini

**Affiliations:** a Micalis Institute, INRAE, AgroParisTech, Université Paris-Saclay, Jouy-en-Josas, 78350, France; b Ifremer, BRM, EM3B Laboratory, F‐44311 Nantes, France

**Keywords:** Aquatic, Genomic, Phylogenetic analysis, Halophilic

## Abstract

Halophilic and psychrophilic marine bacteria are source of interesting bioactive molecules for biotechnology. We report here the whole-genome sequences of two of them, *Pseudoalteromonas sp*. MIP2626 isolated from tropical peeled shrimps and *Psychrobacter sp.* BI730 isolated from deep-sea hydrothermal vent. Sequencing of both genomes was performed by Illumina HiSeq platform (2 × 150 pb). De novo assemblies using Spades v3.9 generated 136 contigs for *Pseudoalteromonas* MIP2626 and 42 contigs for *Psychrobacter* BI730, representing a genome size of 3.9 Mb and 3.2 Mb, respectively. Phylogenetic based on 16S rRNA gene sequence and phylogenomic analyses were reported to compare the new sequences with *Pseudoalteromonas* and *Psychrobacter* representative strains available in the public databases. The genome sequences have been deposited at GenBank under the accession numbers JAATTW000000000 for *Pseudoalteromonas sp.* MIP2626 and JAATTV000000000 for *Psychrobacter sp.* BI730.

Specifications table

**Table:** 

Subject	Molecular biology
Specific subject area	Microbiology and Genomics
Type of data	Draft genome sequences in FASTA format Tables Figures Mtsx tree files (phylogenetic 16S) Newick tree and svg files (phylogenomic)
How data were acquired	We extracted the genomic DNA using phenol-chloroform protocol [1] and whole genome sequencing was performed through the Illumina HiSeq platform.
Data format	Raw and analyzed data
Parameters for data collection	Genomic DNAs were extracted from pure cultures of MIP2626 and BI730 strains.
Description of data collection	Whole-Genome sequencing, assembly, annotation, phylogenetic and phylogenomic comparisons. Genomes were assembled with *de novo* assembly using SPAdes version 3.9 [2] and annotated with Rapid Annotations Subsystems Technology (RAST) [3]. Phylogenetic analysis of 16S rRNA were performed using MEGA 7.0.26 [4]. Multiple Alignment of Conserved Genomic Sequence With Rearrangements (MAUVE 2.4.0) was used for aligned the whole-genome sequences [5]. For the annotation and management of phylogenomic trees, we used iTOL v5 (http://itol.embl.de).
Data source location	*Pseudoalteromonas sp.* MIP2626 was isolated in France from tropical peeled shrimps in 2009 and *Psychrobacter sp.* BI730 from deep-sea hydrothermal vent site in Lau Basin, in the Southwestern Pacific Ocean, in 1989.
Data accessibility	The genome of the MIP2626 strain has been deposited in NCBI database under the Bioproject PRJNA604092, the Biosample SAMN13951749 and the genome accession number JAATTW000000000. The genome of the BI730 strain has been deposited in NCBI database under the Bioproject PRJNA604092, the Biosample SAMN13951813 and the genome accession number JAATTV000000000 All data are available in a public repository: Repository name: Draft-genome sequence data of *Pseudoalteromonas sp.* MIP2626 and *Psychrobacter sp*. BI730 Direct URL to data: http://dx.doi.org/10.17632/kbdfk2d7vy.1

## Value of the data

• The data contribute to describe the genomic diversity of the *Pseudoalteromonas* and *Psychrobacter* species;
• Genome sequence data of halophilic strains from marine origin are useful for comparative genomic analysis and highlight the genetic adaptation of these species in food ecosystems;
• Genome sequences provide new knowledge on *Pseudoalteromonas* and *Psychrobacter* species of biotechnological importance.

## 1 Data Description

*Pseudoalteromonas* and *Psychrobacter* species are Gram-negative moderate halophilic bacteria that could be isolated in different natural ecosystems like soil, salt lake and marine environment [6], but also in salted food such as seafood, processed meat and cheese [7], [8], [9]. As they are psychrophilic bacteria, both genera are interesting for biotechnological applications [10], [11], [12], [13]. In this paper, we present the whole-genome sequences of two strains collected by Ifremer (Nantes, France) from marine samples. The first strain, *Psychrobacter* sp. BI730 was isolated in 1989 from a deep-sea hydrothermal vent site in Lau Basin, in the Southwestern Pacific Ocean, explored during BIOLAU oceanographic cruise. The second one, *Pseudoalteromonas sp.* MIP2626, was isolated in 2009 from tropical peeled shrimps.

**Fig 1:**
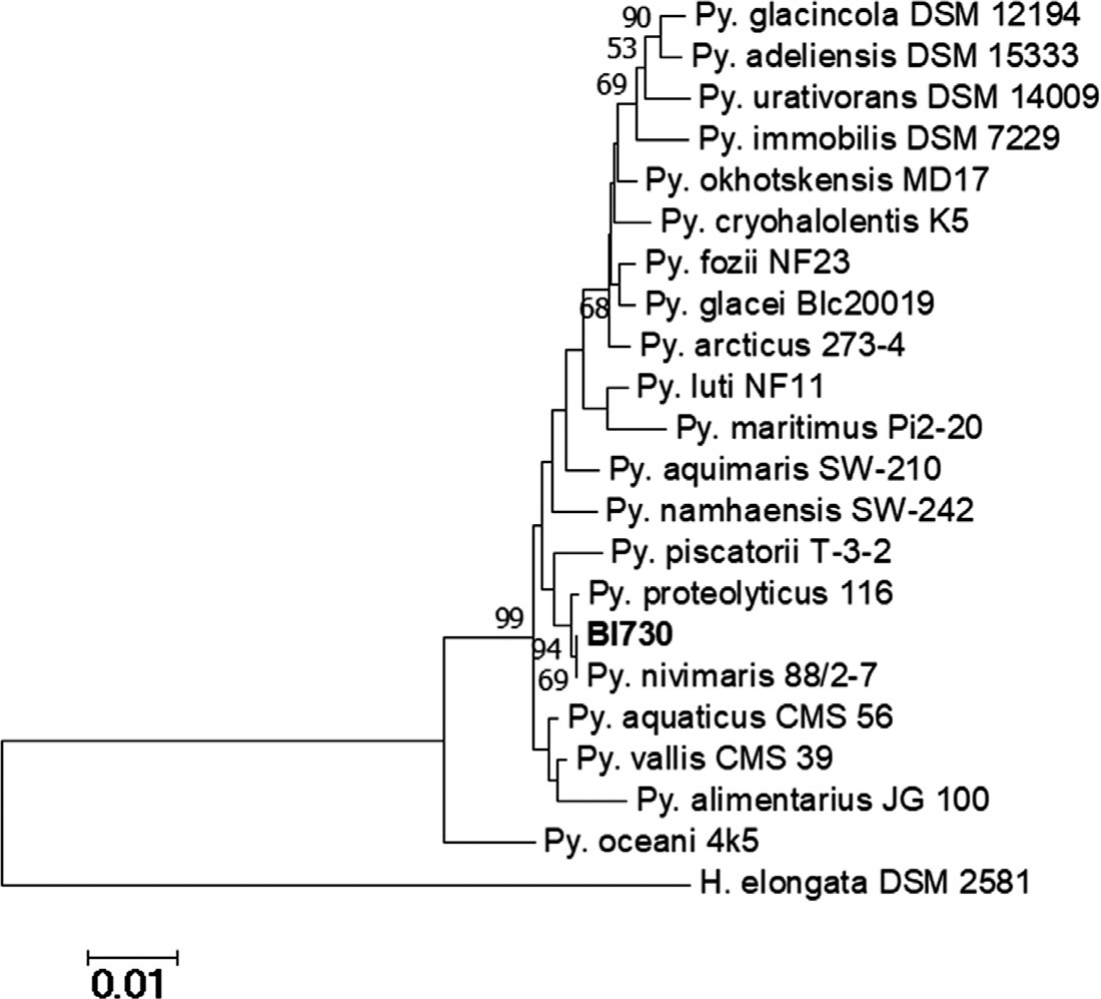
Phylogenetic tree of *Psychrobacter* genus, including *Psychrobacter sp.* BI730 and *Psychrobacter* type strains. The phylogenetic tree is based on 16S rRNA gene alignments obtained by MEGA 7.0.26 software using the neighbor-joining method. *Halomonas elongata* DSM 2581^T^ was used as the outgroup.

**Fig 2:**
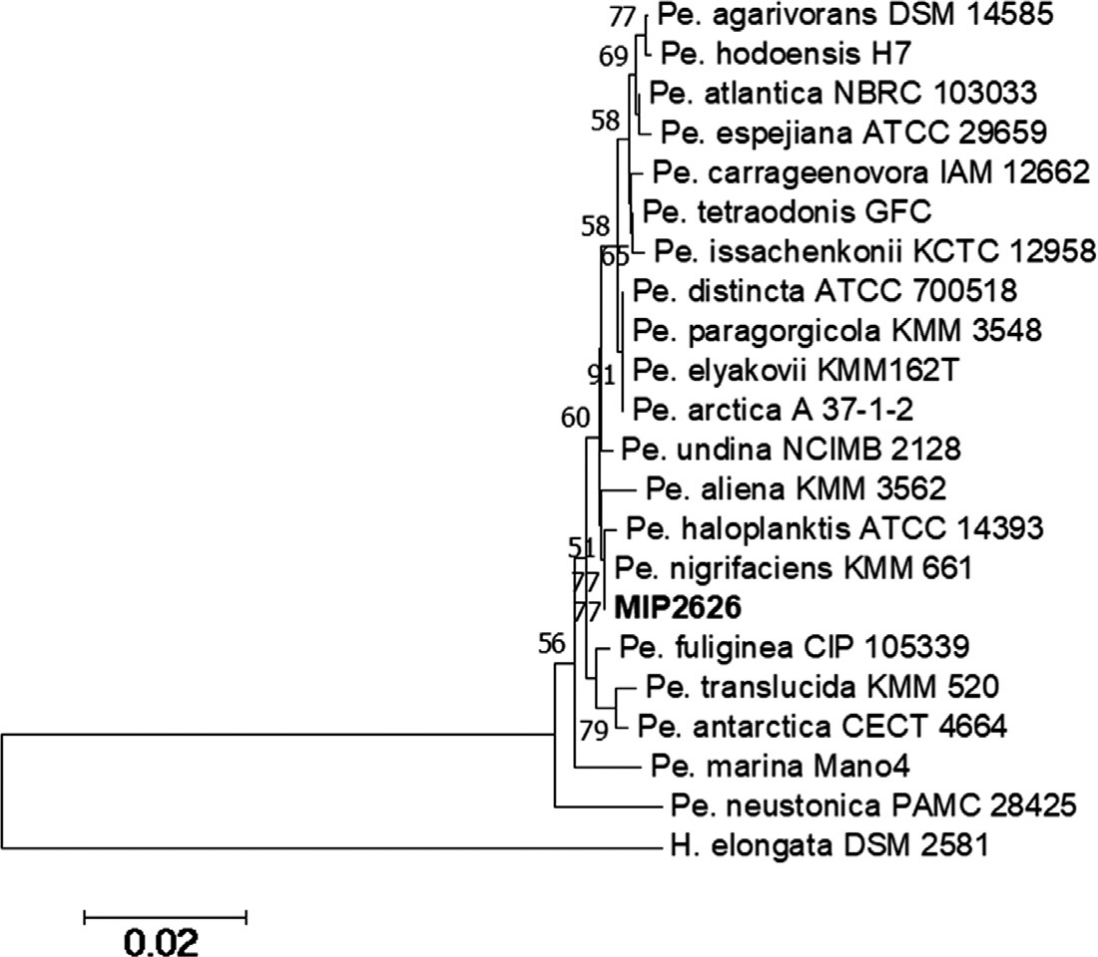
Phylogenetic tree of *Pseudoalteromonas* genus, including the *Pseudoalteromonas sp.* MIP2626 and *Pseudoalteromonas* type strains. The phylogenetic tree is based on 16S rRNA gene alignments obtained by MEGA 7.0.26 software using the neighbor-joining method. *Halomonas elongata* DSM 2581^T^ was used as the outgroup.

Based on the homology of 16S rRNA gene sequences with those of closely type strains (Additional File 1), *Psychrobacter sp*. BI730 shares high similarity with *Psychrobacter nivimaris* 88/2-7^T^ (99.93% for a 1461 bp sequence) (Fig. 1). In the same way, *Pseudoalteromonas sp*. MIP2626 strain appears closely related to *Pseudoalteromonas nigrifaciens* KMM 661^T^ (100% for a 1458 bp sequence) and *Pseudoalteromonas haloplanktis* ATCC 14393^T^ (99.18% for a 1467 bp sequence) (Fig. 2).

Whole-genomes of *Psychrobacter sp*. BI730 and *Pseudoalteromonas sp.* MIP2626 were sequenced through the Illumina HiSeq platform generating respectively 7,118,942 and 4,547,663 paired-end reads (2 × 150 bp) sequences. *De novo* assemblies yielded draft genomes with an average cover of 302.74 for *Psychrobacter sp*. BI730 and 168.00 for *Pseudoalteromonas sp*. MIP2626. Then, the contigs were filtered to keep those of length >300bp and coverage >100. *Psychrobacter sp*. BI730 showed 42 contigs, covering 3,263,843 bp, with 42.7% G+C content and N50 of 207,652 bp. It encodes 2,871 coding sequences classified in 291 subsystems and 46 RNAs. *Pseudoalteromonas sp.* MIP2626 showed 136 contigs, covering 3,988,911 bp, with 40.0% G+C content and N50 of 64,825 bp. It encodes 3,792 coding sequences classified by RAST in 354 subsystems and 97 RNAs.

**Fig 3:**
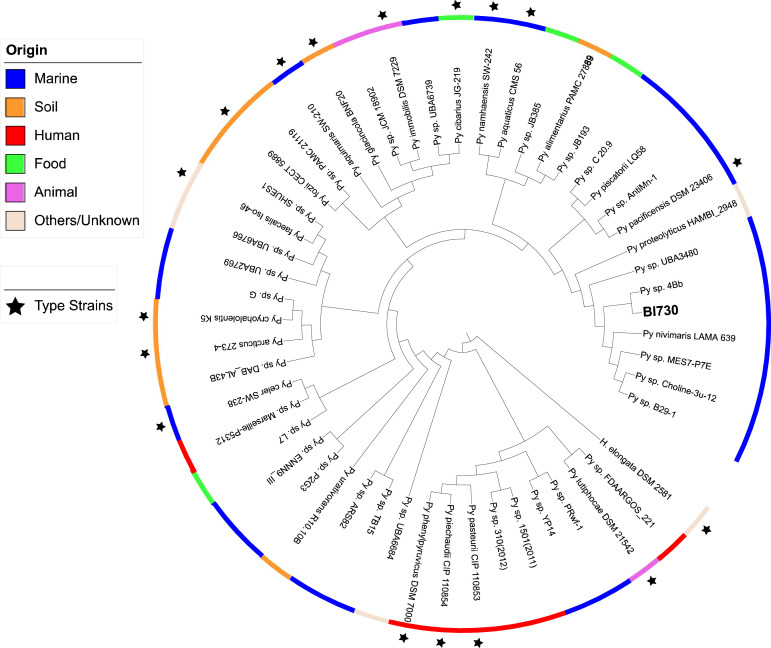
Phylogenomic comparison of *Psychrobacter* strains from diverse origins using the software Mauve 2.4.0 and visualized with iTOL v5. *Halomonas elongata* DSM 2581^T^ was used as the outgroup.

**Fig 4:**
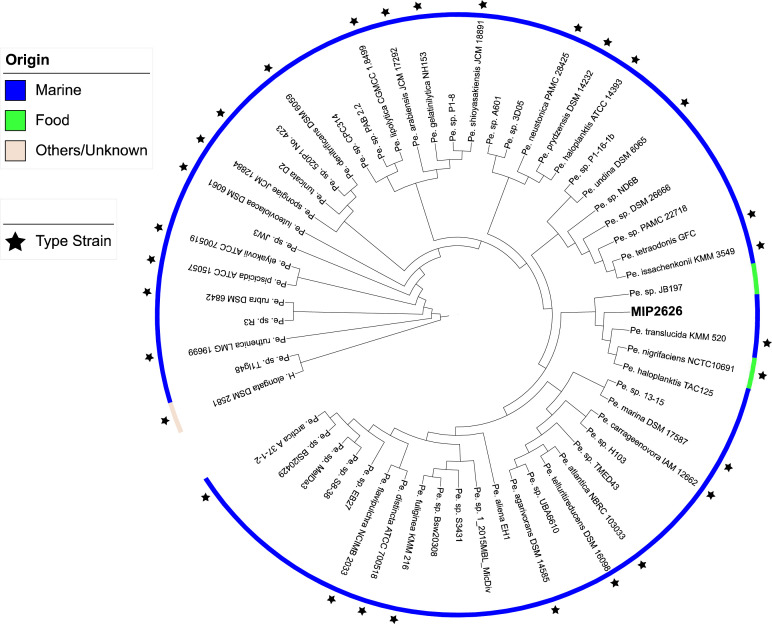
Phylogenomic comparison of *Pseudoalteromonas* strains from marine and food origins using the software Mauve 2.4.0 and visualized with iTOL v5. *Halomonas elongata* DSM 2581^T^ was used as the outgroup.

Phylogenomic comparison based on type strains or reference genomes available in public databases was performed. Further information about the selected reference genomes are shown in Additional File 2. *Psychrobacter sp*. BI730 belongs to a group with several marine strains (Fig. 3). No ANI value over 95 % of BI730 strain with the reference genomes available was observed. *Pseudoalteromonas sp*. MIP2626 is closely related to both food and marine strains (Fig. 4). This strain showed an ANI value higher than 95 % with the type strains *Pseudoalteromonas nigrifaciens* NCTC10691^T^ (97.84 %) and *Pseudoalteromonas translucida* KMM 520^T^ (96.1 %).

## 2 Experimental Design, Materials, and Methods

### 2.1 Genomic DNA extraction

BI730 and MIP2626 strains were grown in Zobell medium at 30°C under aeration and total genomic DNA was extracted using phenol-chloroform protocol [1].

### 2.2 Phylogenetic analysis of 16S rRNA

The 16S rRNA gene was amplified using 27-F (5’-AGAGTTTGATCATGGCTCA-3’) and 1492-R (5’-TACGGTTACCTTGTTACGACTT-3’) primers. Thermal cycling conditions were applied as follow (i) 1 min at 94°C for initial denaturation, (ii) 30 cycles of 1 min at 94°C for denaturation, 0.5 min at 56°C for primer annealing, 1.5 min at 72°C for elongation, and (iii) 5 min at 72°C to ensure final elongation. DNA amplicons were separated on 0.8% agarose gel, purified by using the ExoSAP-IT (Thermo Fisher Scientific, Waltham, Massachusetts, USA) and sent for sequencing to the service provider (Eurofins Genomics, Ebersberg, Germany). Sequences were analyzed to obtain a preliminary taxonomic classification for each isolate.

We elaborated an initial phylogeny of the isolates BI730 and MIP2626 using the closest related 16S rRNA gene sequences generated by EzBiocloud blast (https://www.ezbiocloud.net/). The GenBank accession numbers of these species are shown in Additional File 1. Further phylogenetic analysis were performed using MEGA 7.0.26 software and trees were generated using the Neighbor-Joining algorithm with 1000 bootstrap iterations [4].

### 2.3 Genome sequencing and assembly

Whole genome sequencing was performed through the Illumina HiSeq platform at GATC-Biotech (Konstanz, Germany) The genomes were assembled with *de novo* assembly using SPAdes version 3.9 [2]. Then, the contigs were filtered to keep those of length >300bp and coverage >100. Annotations were produced using the Rapid Annotations using Subsystems Technology server [3].

### 2.4 Phylogenomic analysis

Representative complete genome sequences of *Psychrobacter* and *Pseudoalteromonas* were collected from the NCBI database. Information about these genomes such as isolation origin, Bioproject and BioSample numbers, and Average Nucleotide Identity (ANI) with BI730 and MIP2626 are shown in Additional File 2. ANI values, were determined by JSpeciesWS [14]. *Psychrobacter* and *Pseudoalteromonas* genome sequences selected from the public database were aligned with the BI730 and MIP2626 strains, respectively, using the software Progressive Mauve (version 2.4.0) with the default settings [5]. The phylogenomic trees were visualized with Interactive Tree of Life (iTOL v5) (http://itol.embl.de).

## Declaration of Competing Interest

The authors declare that they have no known competing financial interests or personal relationships which have, or could be perceived to have, influenced the work reported in this article.

## Appendix Supplementary materials

Image, application 1
